# A Scalable Method for Cavity‐Enhanced Solid‐State Quantum Sensors

**DOI:** 10.1002/advs.202517593

**Published:** 2025-12-19

**Authors:** Daniel J. Tibben, Roy Styles, David A. Broadway, Jean‐Philippe Tetienne, Daniel E. Gómez, Philipp Reineck

**Affiliations:** ^1^ School of Science RMIT University Melbourne Australia

**Keywords:** emission enhancement, fluorescent nanodiamond, hexagonal boron nitride, microcavity, quantum sensing

## Abstract

Photoluminescent color centers in diamond and hexagonal boron nitride (hBN) are powerful nanoscale solid‐state quantum sensors that are explored in a plethora of quantum technologies. Methods for integrating them into macroscopic structures that improve their sensitivity and enable their large‐scale deployment are highly sought after. Here, cavity‐enhanced photoluminescence (PL) of fluorescent nanodiamonds (FNDs) and hBN nanoparticles (NPs) embedded in polymer‐based thin‐film optical cavities on the centimeter scale is demonstrated. The cavity resonances efficiently modulate the spectral PL peak position of nitrogen‐vacancy (NV) centers in FNDs across the NV PL spectrum and lead to an up to 2.9‐fold Purcell‐enhancement of the NV PL decay rate. The brightness of hBN NPs increases by up to a factor of three and the PL decay rate is enhanced by up to 13‐fold inside the cavities. Finally, a 4.8 times improved magnetic field sensitivity of 20 nm FNDs is found in thin‐film cavities due to cavity‐enhanced optically detected magnetic resonance contrast and PL brightness. This study demonstrates a low‐cost and scalable method for the fabrication of quantum sensor‐doped thin‐film cavities, which is an important step toward the development of advanced quantum sensing technologies.

## Graphical abstract

Photoluminescent color centres in diamond and hexagon boron nitride are powerful nanoscale solid‐state quantum sensors. Methods for integrating them into macroscopic structures that improve their sensitivity are highly sought after. This study demonstrates a low‐cost and scalable method for the fabrication of quantum sensor‐doped thin‐film cavities, showing cavity‐enhanced photoluminescence in these types of quantum sensors and leading to enhanced sensitivity in quantum sensing experiments.

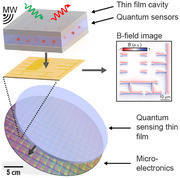

## 1 Introduction

Photoluminescent defects, in diamond, hexagonal boron nitride and silicon carbide with optically addressable spins are vital for a broad range of emerging room‐temperature quantum technologies. The nitrogen vacancy (NV) center in diamond is the most technologically advanced defect and has been used for many imaging and sensing applications,^[^ ^1^, ^2^ ^]^ most of which are based on bulk diamond samples. However, diamond is hard and brittle, relatively expensive, and today's quantum diamond chips are only available up to 4 mm in size. This limits their integration with other materials and their use in large‐area or high‐throughput (e.g., point‐of‐care biomedical devices) applications. Hence, diamond nanoparticles (NPs) on solid substrates^[^ ^3^, ^4^, ^5^, ^6^, ^7^ ^]^ or integrated into other materials^[^ ^8^, ^9^, ^10^, ^11^, ^12^ ^]^ are explored for these applications. However, while fluorescent nanodiamonds (FNDs) containing NV centers offer many potential advantages, their photoluminescence (PL) brightness is generally lower and their spin coherence times shorter than those of NVs in bulk diamond, limiting their sensitivity.

More recently, spin‐active defects in hBN have emerged as a promising 2D material platform for nanoscale sensing.^[^ ^13^ ^]^ Unlike diamond, many as‐synthesized hBN materials from NPs to bulk crystals, contain a range of quantum emitters with PL from the violet to the near‐infrared spectral region in the same material.^[^ ^14^ ^]^ However, the PL properties of individual particles can vary greatly^[^ ^15^ ^]^ and one defect that can be controllably created in hBN,^[^ ^16^ ^]^ the boron vacancy $V_{B}^{-}$,^[^ ^17^, ^18^ ^]^ is very dim and has not been observed as a single photon emitter.

**Figure 1:**
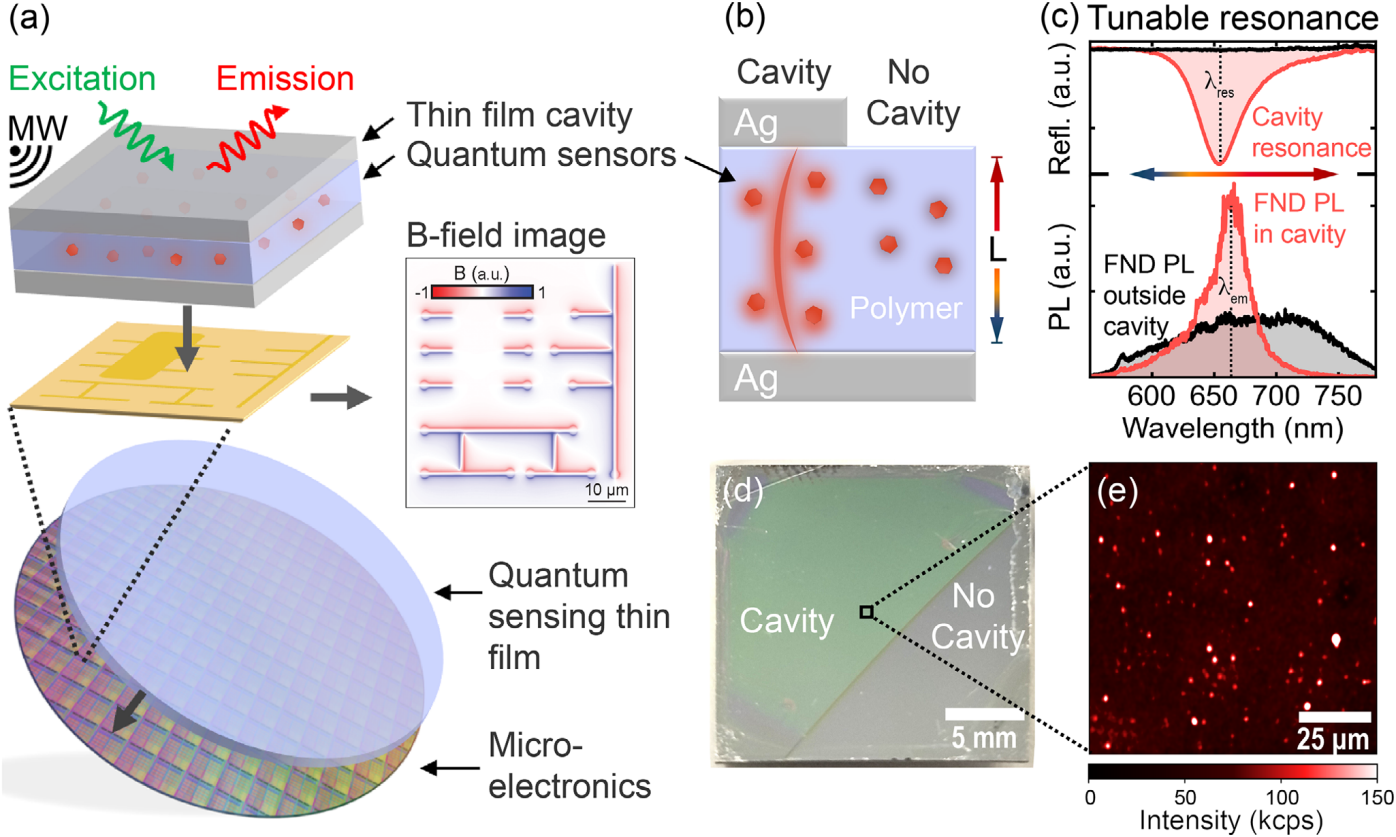
Application and design of quantum sensor‐doped microcavity thin‐films— a) Schematic illustration of the application of our thin‐films to the magnetic imaging of currents in a microcircuit via cavity–enhanced quantum sensors. b) Schematic of the cavity and control ('no cavity') devices with embedded quantum sensor particles. The cavity resonance energy is controlled by the polymer layer thickness *L*, allowing tuning of the spectral position of the cavity resonance. c) Typical reflectivity spectrum of the cavity resonance (top) and PL spectra of FNDs inside (red trace) and outside the cavity (black trace). d) A photograph of a typical thin‐film microcavity‐coated substrate, with clearly distinguishable cavity (green) and no cavity (clear) regions. e) Confocal PL image of the microcavity in panel (c), showing PL from FNDs uniformly dispersed throughout the thin‐film.

Hence, methods for controlling and enhancing FND and hBN PL properties are highly sought after. One approach is to use optical cavities for Purcell‐enhanced PL, which has been explored for emitters in diamond.^[^ ^19^, ^20^ ^]^ and hBN.^[^ ^21^, ^22^ ^]^ For diamond, this has been explored for individual FNDs in photonic crystal cavities,^[^ ^23^, ^24^, ^25^, ^26^ ^]^ in metamaterial photonic cavities,^[^ ^27^ ^]^ in open cavities,^[^ ^28^, ^29^ ^]^ and photonic crystal cavities in bulk diamond.^[^ ^30^, ^31^, ^32^, ^33^ ^]^ For hBN, this enhance ment approach has been explored in planar Fabry–Pérot–based cavities,^[^ ^34^, ^35^, ^36^ ^]^ open cavities,^[^ ^37^ ^]^ plasmonic cavities,^[^ ^38^, ^39^ ^]^ micronanoresonators,^[^ ^40^, ^41^, ^42^ ^]^ and photonic crystal cavities.^[^ ^43^, ^44^, ^45^, ^46^, ^47^ ^]^ However, to date, no straightforward and scalable method for the fabrication of thin‐film cavities containing nanoscale quantum sensors has been reported. Wafer‐scale quantum sensor‐doped thin‐film cavities can enable, for example, magnetic imaging of currents in microelectronics^[^ ^48^ ^]^ as illustrated in **Figure** **1**a.

Here, we demonstrate cavity‐enhanced PL of FND and hBN NP quantum sensors embedded in polymer‐based thin‐film cavities. We report a straightforward and scalable method for the fabrication of quantum sensor‐doped thin‐film microcavities on the centimeter scale. The PL properties of FNDs and hBN NPs are investigated as a function of the spectral position of the cavity resonance across the visible and near‐infrared spectral range. The cavity resonances efficiently modulate the spectral PL peak position of NV centers in FNDs across the NV PL spectrum and lead to an up to 2.9‐fold enhancement of the NV PL decay rate compared to FNDs outside the cavity. The hBN NP PL brightness increases by up to a factor of three and the PL decay rate is enhanced up to 13‐fold inside the cavity thin‐films. By comparing experimental and theoretical Purcell enhancement factors, we conclude that Purcell enhancement causes the observed modulation of NV PL and that Purcell enhancement alone cannot explain the strong PL brightness and decay enhancement of hBN NP PL. Finally, we demonstrate an up to 4.8 times improved magnetic field sensitivity of 20 and 100 nm FNDs in thin‐film cavities due to a cavity‐enhanced optically detected magnetic resonance (ODMR) contrast.

## 2 Results and Discussion

Low‐cost device‐integrated quantum sensors, such as the thin‐film of quantum sensors depicted in Figure 1a, are critical to their widespread adoption in commercial applications. To this end, we designed a series of polymer‐based Fabry–Pérot microcavity devices with embedded FNDs and hBN NPs and explored the cavity–modified optical properties of the quantum sensors by comparing their optical properties inside and outside the cavity on the same device, as illustrated in Figure 1b. The devices comprised of a silver mirror on either a Si wafer or quartz substrate as the base layer, followed by a polymer thin‐film doped with either FNDs or hBN NPs, and capped with a semi‐transparent top mirror. This design ensures the formation of confined optical cavity modes that interact with the quantum sensors embedded in the polymer layer. The resonance wavelength of the modes can be controlled via the polymer thickness *L*. As an example, Figure 1c, top, shows the reflectance spectrum of a cavity with a polymer layer thickness of 159 nm and a typical PL spectrum of an FND inside this cavity (bottom). The cavity shows a pronounced resonance peak at λ_*res*_ = 650 nm. This resonance leads to a spectrally narrower cavity‐enhanced FND PL peaking at λ_*em*_ = 664 nm compared to the FND PL outside the cavity (Figure 1c, black trace), which we will investigate in detail in the following.

To fabricate the microcavities, we first deposit a fully reflective 100 nm Ag layer on Si via electron beam evaporation physical vapor deposition to create flat, uniform substrates for spin coating. For the FND cavities, ∼120 nm FNDs (see Experimental Section for details) containing 1 ppm of NV centers were dispersed at a concentration of 0.5 mg mL^−1^ in a 1:3 water:1‐propanol solution containing polyvinyl pyrrolidone (PVP) at 2.5 wt%. This suspension was spin‐coated at speeds from 600 to 2600 rpm to produce FND‐doped polymer thin‐films ranging in thickness from 107 to 210 nm. Finally, a semi‐transparent Ag layer was deposited via electron beam evaporation to complete the microcavity. For hBN, this fabrication protocol was repeated using as‐received hBN particles (Graphene supermarket, 70 nm particle size) dispersed at a concentration of 0.3 mg mL^−1^ in a solution containing polymethyl methacrylate (PMMA) in chlorobenzene at 3.0 wt.%. These particles are known to contain a range of photoluminescent spin‐active defects that emit from the violet to near‐infrared spectral region.^[^ ^14^ ^]^

Figure 1d shows a photograph of a 20 × 20 mm Si‐wafer substrate, half of which is coated with a complete FND‐doped microcavity (left), while the other half lacks the top Ag mirror for control experiments (right). The light absorption by the resonant cavity mode produces a green tint in the cavity region, which is not present in the no‐cavity region. Figure 1e shows a confocal PL image of the FND microcavity, showing evenly distributed FND particles throughout the field of view. For both FND and hBN microcavities, we fabricated a suite of devices with fundamental optical modes spanning the spectral region from 500 to 800 nm, covering the spectral emission range of the NV center in FNDs and the hBN emitters. Figure S1a,b (Supporting Information) shows the normal‐angle reflectance spectra for the FND and hBN microcavities, respectively, clearly showing reflectance anti‐peaks with reflectivity decreases of 60–80% at the predicted resonance mode energies. All experiments were performed at room temperature.

**Figure 2:**
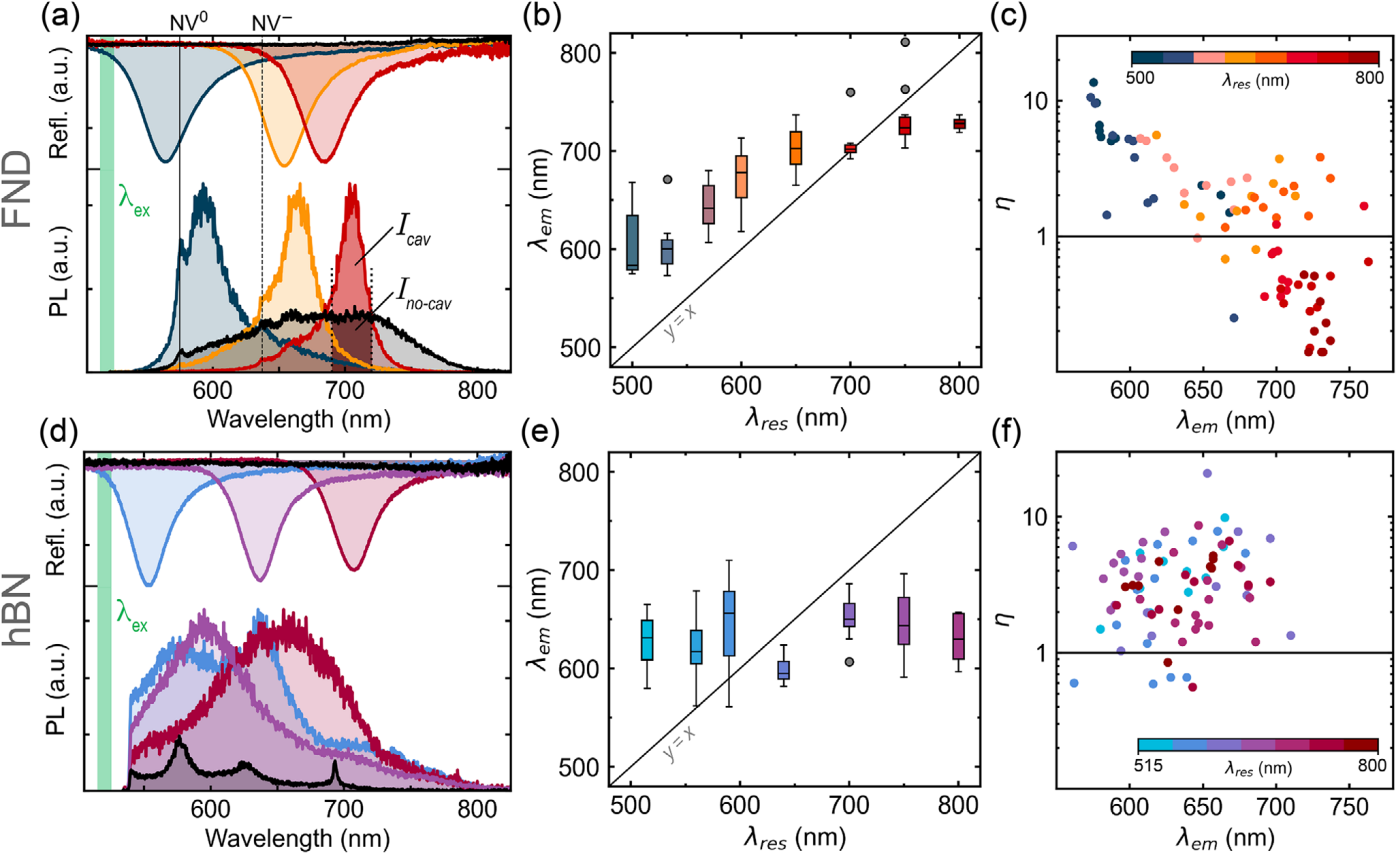
a,d) Thin‐film cavity reflectivity spectra (top, colored traces) and corresponding typical PL spectra of FND (a) and hBN NP (d) quantum sensors embedded in the cavity (bottom, colored traces). Black traces at the top of a) and d) show the no‐cavity reflectivity and no‐cavity PL spectra of FNDs (a) and hBN (d) at the bottom. The green bar on the left indicates the excitation wavelength *λ*_*ex*_ = 520 nm. b,e) Box and whisker plot of *λ*_*em*_ as a function of *λ*_*res*_ for all investigated FNDs (b) and hBN NPs (e). The horizontal dash in the box represents the average, the edges of the box the upper and lower quartiles, and the whiskers the minimum and maximum values of *λ*_*em*_. Statistical outliers (1.5 × interquartile range) are in gray. Sample size *n* = 10 for each cavity device. c,f) Scatter plot of cavity enhancement factor η (see main text for details) as a function of *λ*_*em*_ for individual FNDs (c) and hBN particles (f), where the color of each dot indicates *λ*_*res*_ of the cavity.

**Figure 3:**
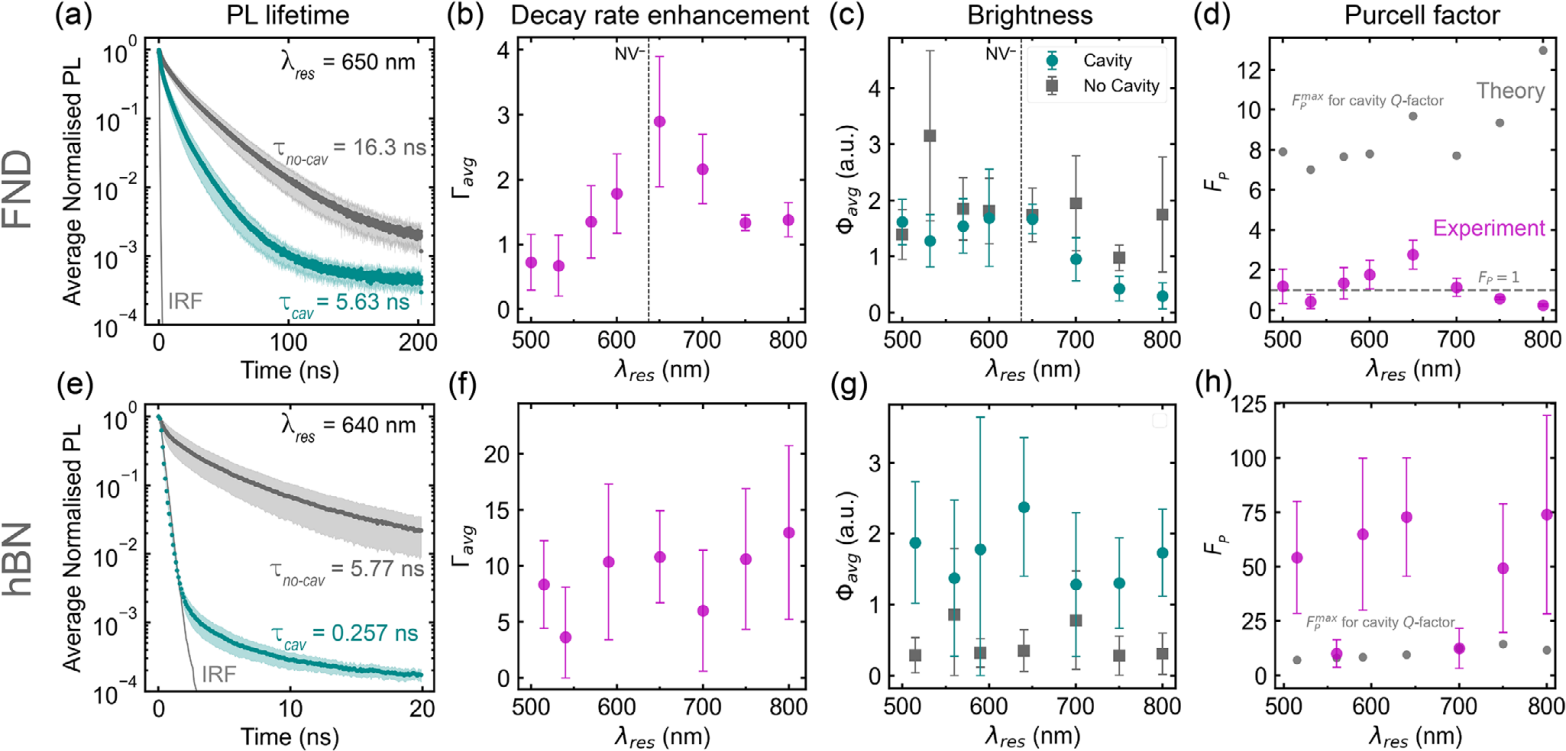
Emission enhancement of FNDs and hBN NPs inside thin‐film cavities— a,e) Averaged time‐resolved PL decay traces of FNDs (a) and hBN NPs (e) inside (green traces) and outside (grey traces) the cavities. The shaded envelopes show the standard deviation between individual measurements and the black dotted line the instrument response function (IRF). b,f) Average emission rate enhancement *Γ*_*avg*_ for FND (b) and hBN (e) cavities as a function of cavity resonance wavelength *λ*_*res*_. c,g) Average total PL brighness Φ for FND (c) and hBN (f) cavities as a function of cavity resonance wavelength *λ*_*res*_. d,h) Experimental (pink markers) and theoretical (grey markers) Purcell enhancement factor *F*_*P*_ for FND (d) and hBN (h) cavities as a function of cavity resonance wavelength *λ*_*res*_. Sample size *n* = 10 for each cavity and no‐cavity device, at each resonance condition.

First, we investigated the effect of the cavities on the FND and hBN NP spectral PL characteristics and PL brightness changes in the spectral region of the cavity resonance (**Figure** **2**). We used a confocal PL microscope with 5 ps pulsed 520 nm laser excitation and collected PL above 540 nm in all experiments (see Experimental  for details). We collected PL spectra and time‐resloved PL decay traces for FNDs and hBN NPs inside and outside cavities, which are analyzed and discussed in detail in the following text and in Figures 2 and 3.

Typical reflectance and PL spectra for three FND cavity devices are depicted in Figure 2a, where the reflectance anti‐peak, a signature of the resonant cavity mode energy, is color‐coded to the corresponding PL spectrum from FNDs inside the cavity. The “no‐cavity” control device showed no absorption in the reflectance profile (black trace), indicating the absence of a cavity resonance and the FND PL spectrum (Figure 2a, black trace) shows a typical NV emission profile with contributions from the NV^0^ and NV^−^ charge states. The PL spectra of FNDs inside the cavity (Figure 2a, colored traces) were strongly modulated and significantly narrower, with the PL peak position λ_*em*_ shifting with the cavity resonance peak position *λ*_*res*_. The characteristic NV^0^ and NV^−^ zero phonon lines at 575 and 637 nm, respectively, are clearly visible in all spectra, confirming the NV center as the origin of the PL.

While each cavity resonance shown in Figure 2a is representative of the entire thin‐film microcativy, the PL spectra were acquired from individual FNDs. The spectra in Figure 2a suggest that *λ*_*em*_ is red shifted from *λ*_*res*_ by 10–20 nm. To determine if this shift is typical for most FNDs in all thin‐film cavities, we acquired PL spectra from 10 individual FNDs in each cavity. Figure 2b shows a box and whisker plot of *λ*_*em*_ as a function of *λ*_*res*_ for all investigated FNDs. The horizontal dash in each box represents the average, the edges of the box the upper and lower quartiles, and the whiskers the minimum and maximum values of *λ*_*em*_. Statistical outliers (1.5 × interquartile range) are in gray. The average *λ*_*em*_ linearly increases with *λ*_*res*_ for *λ*_*res*_ values between 525 and 650 nm and only slightly increases for *λ*_*res*_ > 650 nm. In the *λ*_*res*_ range of 500–650 nm, *λ*_*em*_ is on average red‐shifted by more than 50 nm, with a maximum shift of 80 nm for *λ*_*res*_ = 500 nm. Above 700 nm, *λ*_*em*_ is slightly blue‐shifted relative to *λ*_*res*_. Overall, Figure 2b demonstrates that the average spectral emission peak position of FND NV PL can be reliably tuned by the cavity resonance.

The discrepancy between *λ*_*em*_ and *λ*_*res*_ is likely caused by a local modification of the effective refractive index *n*_*eff*_ of the polymer thin‐film by the FND particles. The reflectance spectra in Figure 2a represent averages over an 100 × 100 µm^2^ area of the cavity and only a small fraction of this area contains FNDs. The PL spectra on the other hand are acquired using a focused laser beam with a diameter of ca. 300 nm, where an FND covers a significant fraction of the illuminated area. In this area and associated thin‐film volume, the FND increases *n*_*eff*_ of the thin‐film due to the higher refractive index of diamond (*n* = 2.4) compared to pure PVP (*n* = 1.5), leading to a red‐shift in the cavity resonance wavelength.

We then investigated the FND PL intensity enhancement in the spectral region of the cavity resonance. We define this spectral cavity enhancement $\eta =I_{cav}/I_{no-cav}^{avg}$, for constant laser power impinging on the device, where *I_*cav*_* and *I* 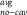 are the integrated PL intensities ±15 nm around *λ*_*em*_ of individual FND PL spectra inside the cavity and the average PL spectrum of FNDs outside the cavity, respectively (see Figure S2 for details, Supporting Information). Figure 2c shows a scatter plot of η as a function of *λ*_*em*_ for individual FNDs, where the color of each dot indicates λ_*res*_ of the cavity. We find the strongest enhancement of up to *η* = 10 for the cavity with *λ*_*res*_ = 500 nm and *λ*_*em*_ emission peak positions between 575–600 nm, close to the ZPL of NV^0^ at 575 nm. On average, η drops significantly with increasing *λ*_*em*_ and *η* varies significantly between individual particles with most enhancement values between 1 and 3 for *λ*_*em*_ between 625 and 700 nm. Above *λ*_*em*_ = 700 nm, FNDs show η values above and below 1, indicating that both PL enhancement and suppression occur in this region.

In principle, both the excitation beam (*λ*_*ex*_ = 520 nm) and NV center PL can couple to the cavity resonance mode. Our experiments cover three cases. 1) The excitation beam and NV PL couple to the cavity resonance (*λ*_*res*_ = 500 − 570 nm), and we observe the strongest spectral emission enhancement of up to *η* = 10. 2) Mostly NV PL couples to the cavity resonance (*λ*_*res*_ = 600 − 700 nm). Here, the cavity enhancement is still present (*η* > 1) but reduced, since most excitation light is reflected (see red and orange traces in Figure 2a). 3) NV PL only weakly couples to the cavity resonance (*λ*_*res*_ > 700 nm). The NV PL decreases above 700 nm and most excitation light is reflected resulting in suppression of NV PL inside the cavity compared to outside the cavity in most cases (see red markers in Figure 2c), likely due to optical losses in the top mirror.

We then performed the same analysis for hBN NPs. The hBN particles studied here contain a range of carbon‐related defects,^[^ ^14^ ^]^ whose spectral PL characteristics vary greatly between individual particles, with emission from 550–800 nm upon 520 nm excitation (see Figure S3 for examples, Supporting Information). Figure 2d shows the PL spectrum of an hBN particle outside the cavity (black trace) compared to spectra of hBN particles in three different cavities (colored traces). Unlike for FNDs, there is no systematic correlation between *λ*_*res*_ and *λ*_*em*_. Due to the great variation in hBN PL spectral characteristics, even the identification of a single emission peak position is nontrivial (see Figure S4 for details, Supporting Information). Figure 2e shows a box and whisker plot of *λ*_*em*_ as a function of *λ*_*res*_ for all investigated hBN particles. It confirms that *λ*_*em*_ of most hBN particles inside the cavity is between 600 and 650 nm, irrespective of *λ*_*res*_, with a large spread of *λ*_*em*_ values between 570 and 700 nm. Interestingly, we nonetheless observe that the PL of more than 90% of hBN particles in cavities is enhanced 2–10 times compared to particles outside the cavity (Figure 2f). The hBN particles generally appeared slightly larger than FNDs in confocal PL images (see Figure S5, Supporting Information) suggesting hBN NP aggregation. Due to their larger size, the aggregates may have been in closer proximity to the top and bottom silver mirror, leading to surface plasmon‐related enhancements that are independent of the cavity resonance.

### 2.1 FND Emission Rate Enhancement

Having established the effect of the cavity on the quantum sensors' spectral PL properties, we investigated the effect of the cavities on the PL lifetime and total PL brightness to quantify the cavity‐induced modulation of FND and hBN PL. Figure 3a shows the average time‐resolved PL decay traces of ten FNDs inside and 10 FNDs outside the cavity with *λ*_*res*_ = 650 nm. The shaded envelope represents the standard deviation between individual measurements. FNDs inside the cavity show a significantly shorter PL lifetime, of *τ*_*cav*_ = 5.63 ns compared to *τ*_*no* − *cav*_ = 16.3 ns for FNDs outside the cavity. These lifetimes were determined by an amplitude‐weighted average of a double exponential fit to the decays to accurately account for both short‐ and long‐lived components, as defined in the Supporting Information. To quantify the cavity‐induced reduction in the PL lifetime we investigated the decay rate enhancement *Γ*, which we define as the ratio of the observed decay rate γ inside and outside the cavity.

(1) $$\Gamma =\frac{\gamma_{cav}}{\gamma_{no-cav}^{avg}},$$

where the decay rate *γ* = *τ* ^−1^ and γ_*cav*_ and $\gamma_{no-cav}^{avg}$ are the decay rate of ten individual FNDs inside the cavity and the average decay rate of ten FNDs outside the cavity, respectively. Figure 3b shows the average decay rate enhancement *Γ*_*avg*_ of ten investigated FND cavities per cavity device as a function of the cavities' spectral resonance peak position *λ*_*res*_. Error bars represent the standard deviation between individual measurements. Cavities with *λ*_*res*_ between 575 and 800 nm exhibit a *Γ*_*avg*_ > 1, with a peak enhancement of 2.9 for *λ*_*res*_ = 650 nm. Only cavities with resonances below 600 nm show no statistically significant change in the decay rate. Hence, all cavity devices with resonances in the spectral region where the NV emits, i.e. 570–800 nm, show a decay rate enhancement. The fact that we observe strong spectral PL enhancement *η* for cavities with resonances below 600 nm (Figure 2c), where no emission rate enhancement is present (Figure 3b) suggests that cavity enhancement of the excitation beam causes the spectral PL enhancement in this spectral region.

The measured PL decay rate γ is the sum of radiative *γ* ^*rad*^ and non‐radiative decay *γ* ^*nr*^ rates. To identify whether the observed increase in γ is the result of an increase in *γ* ^*rad*^, *γ* ^*nr*^ or both, we investigated the total PL brightness Φ of FNDs, which is proportional to the PL quantum yield *γ* ^*rad*^/*γ* ^*rad*^ + *γ* ^*nr*^. We determined *Φ* for FNDs inside and outside the cavity as the total area under the PL spectra for each FND. Figure 3c shows *Φ_*avg*_* for all devices as a function of *λ*_*res*_ for FNDs inside and outside the cavity. Markers represent the brightness averaged over 10 FNDs per sample and the error bars the standard deviation between individual FNDs. In half of the devices, FNDs inside and outside the cavity exhibit a similar brightness, with some devices showing a roughly twofold decrease (*λ*_*res*_ = 525 nm and *λ*_*res*_ = 700 nm and above). Importantly, the FND brightness is the same inside and outside the cavity (within the standard deviation) for three devices with *λ*_*res*_ values of 575–650 nm, for which we see significant decay rate enhancements. Given the observed NV decay rate enhancement of 2.9 observed for *λ*_*res*_ = 650 nm, assuming no optical losses in the Ag top mirror, and an NV quantum yield of 0.5,^[^ ^49^ ^]^ this would suggest that both *γ* ^*rad*^ and *γ* ^*nr*^ increase by a factor of 2.9, which would lead to no change in the NV quantum yield and brightness. However, considering light absorption in the Ag mirror is present but difficult to quantify in our cavities, an increase in the radiative rate is likely dominant.

To investigate whether the observed enhancement can be understood in terms of a Purcell enhancement, we determined an experimental Purcell enhancement factor *F*_*P*_^[^ ^50^ ^]^ and compared it to a theoretical enhancement factor $F_{P}^{th.}$. These are defined as

(2) $$F_{P}=\frac{\Phi_{cav}}{\Phi_{no-cav}}\frac{\gamma_{cav}}{\gamma_{no-cav}}$$

where *Φ*_*cav*_ and *Φ*_*no* − *cav*_ are the average PL brightness inside and outside the cavity, respectively, (Figure 3c) and *γ*_*cav*_ and *γ*_*no* − *cav*_ are the observed PL decay rates inside and outside the cavity, respectively. The theoretical Purcell factor can be estimated for these devices as

(3) $$F_{P}^{th.}≃\frac{6Q}{\pi^{2}}$$

where *Q* is the cavity quality factor (see Supporting Information for further details).

Figure 3d shows the *F*_*P*_ and $F_{P}^{th.}$ for all FND cavities as a function of *λ*_*res*_. Most cavities exhibit a theoretical Purcell enhancement of $F_{P}^{th.}=7.5\pm 1.0$. The cavity with *λ*_*res*_ = 650 nm has a slightly higher $F_{P}^{th.}$ of 9.7, coinciding with the highest observed experimental *F*_*P*_ of 2.9 in the spectral region of the NV^−^ ZPL. In the spectral region above 700 nm where the NV PL begins to drop off, $F_{P}^{th.}$ slightly increases while *F*_*P*_ decreases. In all cases *F*_*P*_ is significantly lower than $F_{P}^{th.}$, which is expected since cavity losses, e.g., in the Ag mirrors, are not considered in our model. However, the three devices with resonances from 575 to 650 nm, for which the highest decay rate enhancement was observed, all show average *F*_*P*_ > 1, suggesting that a Purcell enhancement of the NV radiative rate causes the observed enhancements.

### 2.2 hBN Emission Rate Enhancement

We now focus on the hBN cavities, the results of which are presented in the bottom row of Figure 3. Unless noted otherwise, the hBN data were analyzed as described for FNDs above. Figure 3e shows the average time‐resolved PL decay traces of hBN particles inside and outside the *λ*_*res*_ = 640 nm cavity up to 20 ns after the excitation pulse, i.e., on a timescale ten times shorter than for FNDs in Figure 3a. While hBN particles outside the cavity show an average PL lifetime of *τ*_*no* − *cav*_ = 5.77 ns (amplitude‐weighted average of a double exponential fit), >99% of photons in the cavity are emitted within the first 2 ns of the decay and faster than our instrument response function, suggesting a dominant PL lifetime *τ*_*cav*_ < 0.5 ns. Only a very small fraction (<0.1%) of photons are emitted after 3 ns, with a lifetime of 6.82 ns, similar to *τ*_*no* − *cav*_.

Figure 3f shows the resulting decay rate enhancement *Γ*_*avg*_ for all hBN devices as a function of *λ*_*res*_. We find significant decay rate enhancements for all devices with average values for different devices ranging from 4 to 13 and also strong variations between individual particles within the same device. Figure 3g shows the corresponding average hBN brightness as a function of *λ*_*res*_. Unlike for FNDs, we find a systematic increase in PL brightness for hBN particles inside the cavity compared to outside and no systematic dependence on *λ*_*res*_. We also observed that the hBN NPs inside the cavity showed pronounced fluctuations in PL intensity over time. These fluctuations were also observed in real‐time in steady‐state widefield measurements and is a behavior not observed in the no‐cavity control devices. This blinking may arise from transient switching of defect centers between radiative and non‐radiative states.^[^ ^51^ ^]^ Coupling to the planar metallic microcavity may enhance these effects through resonant interactions, including via Purcell effects and plasmonic coupling, where fluctuations in the charge state of the emitter may be amplified in the cavity, leading to the temporally unstable blinking observed in these measurements. These observations indicate that the cavity not only modifies spectral features but also reveals dynamic emission behaviors that are absent in uncoupled nanoparticles.

Figure 3h shows the Purcell enhancement factors *F*_*P*_ and $F_{P}^{th.}$ as a function of cavity *λ*_*res*_. Due to the combination of a strong PL decay rate enhancement and PL brightness enhancement, we obtain *F*_*P*_ values of up to 75, which is an order of magnitude higher than the highest theoretical enhancement $F_{P}^{th.}$. We therefore conclude that Purcell enhancement alone cannot explain the observed extreme modulation of hBN PL in our cavities.

Several mechanisms may explain our observations. Both the excitation beam and the hBN PL can be enhanced by cavity resonances. Since we don't observe a clear dependence of emission enhancement on λ_*res*_, it is unlikely that cavity resonances are the main cause for the enhancement. The close proximity of the hBN particles to the top and bottom silver mirror may cause surface plasmon‐related enhancements. Direct electrical contact between hBN particles and one of the mirrors can also lead to a fast light‐induced redistribution of charges that may explain the fast PL decay. Lastly, a local change in cavity geometry due to the formation of hBN aggregates could alter the cavity properties around particles and may focus light onto hBN particles independent of the polymer layer thickness. Future studies will investigate the origin of the observed PL modulation.

### 2.3 Enhancement of Quantum Sensing

Finally, we investigated whether the observed enhancement of FND and hBN PL inside cavities improves their quantum sensing performance. We first focused on FNDs. We employed a custom built wide‐field quantum sensing microscope^[^ ^52^ ^]^ (Figure S6, Supporting Information) to acquire ODMR spectra for FNDs inside the cavities investigated in the previous section as a function of cavity resonance λ_*res*_. See Experimental Section for full experimental details. These devices were fabricated on Si wafer substrates, which strongly absorb microwaves. Hence, microwaves were delivered via a loop antenna from the top‐mirror side of the cavity, which made a direct comparison between FNDs inside and outside the cavity challenging due to top Ag mirror MW absorption only being present on the cavity side. However, it allowed us to investigate the ODMR contrast for all cavity resonances. We find the highest ODMR contrast of 4.4% for the cavity with *λ*_*res*_ = 650 nm, which gradually drops to approximately 2% for resonance wavelengths of 500 and 800 nm (see Figure S7, Supporting Information).

We were unable to acquire ODMR spectra for the hBN thin‐film cavities. In general, the positive ODMR contrast of the spin pair emitters in the hBN NPs investigated here is below 1% even under optimized MW delivery conditions.^[^ ^14^, ^53^ ^]^ Hence, one possible explanation is that the MW field inside our cavities was too low to efficiently drive the hBN spin transitions. For the hBN emitters investigated here, the MW‐induced spin mixing that is the basis of ODMR occurs in a metastable state.^[^ ^53^ ^]^ Hence, another possible explanation is that the PL decay observed for most hBN NPs inside the cavity became so fast, i.e., the excited state lifetime so short, that transitions to the metastable state were very inefficient resulting in a negligible population of the metastable state. Lastly, the decrease in hBN PL stability over time increased the measurement noise in ODMR spectra.

**Figure 4:**
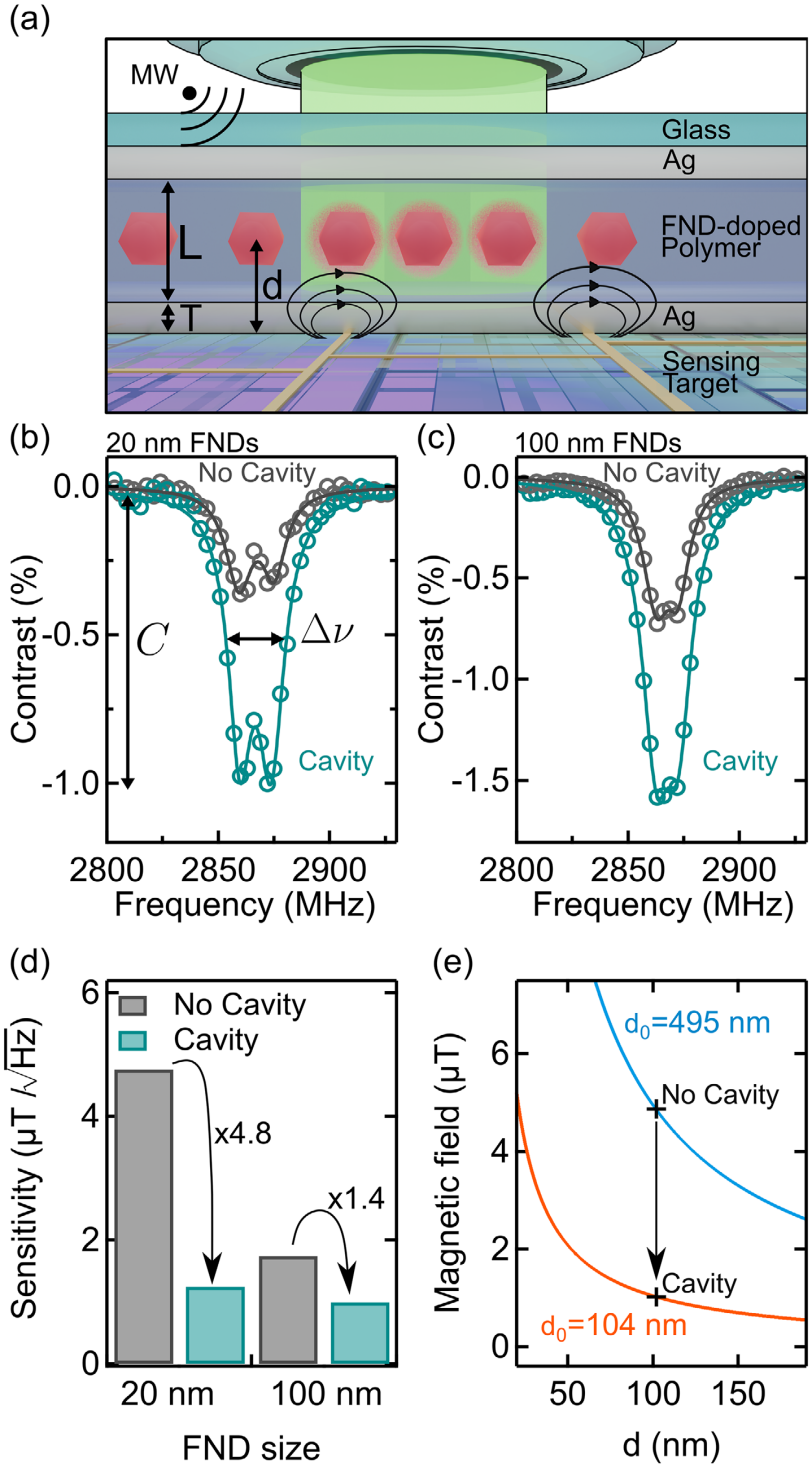
Cavity‐enhanced magnetic sensing— a) Schematic illustration of the experimental setup employed to estimate the magnetic field sensitivity of 20 and 100 nm FNDs. The microelectronic circuit sensing target underneath the FND cavity is an envisaged use case for FND thin‐film cavity‐enhanced magnetic imaging. b,c) Average ODMR spectra of 20 nm (b) and 100 nm (c) FNDs inside and outside the cavity, from 3 separate 27 × 27 µm^2^ regions of interest. d) Magnetic field sensitivity *S* for 20 nm and 100 nm FNDs inside and outside the cavity. e) Magnetic field *B* at the location of the nanoscale sensor as a function of its distance *d* from a source (e.g., an electrical current in a microchip) for our cavity (orange trace) and a hypothetical thicker cavity (blue trace). The black markers indicate the smallest B‐field detectable per $\sqrt{Hz}$ for 20 FNDs inside and outside the cavity.

Based on these results, we focused on the FND cavities, selected the *λ*_*res*_ = 650 nm cavity for further analysis and fabricated devices on quartz substrates that allow MW delivery and imaging through the substrate and hence a direct comparison between FNDs inside and outside the cavity. **Figure** **4**a illustrates the experimental setup, the geometry of the FND cavity, and an envisaged use case, where the FND cavities are positioned on top of a microelectronic circuit to image the local current in the circuit via the generated magnetic field. In the following, we estimate the magnetic field sensitivity of our devices based on ODMR spectra and the PL brightness for two FND particle sizes.

We fabricated one device with 20 nm FNDs and one with 100 nm FNDs (Adamas Nanotechnologies, USA) to also investigate if the much dimmer PL, mostly from the NV^0^ charge state of the 20 nm FNDs^[^ ^54^ ^]^ can be enhanced for ODMR‐based quantum sensing. Reflectance spectra for these devices is shown in Figure S8 (Supporting Information). Figure 4b,c shows ODMR spectra for 20 and 100 nm FNDs, respectively, inside and outside the cavity, with the characteristic NV^−^ resonance at 2.87 GHz. The ODMR contrast increases by a factor of 2.8 (20 nm FNDs) and 2.2 (100 nm FNDs) while the full‐width half‐maximum (FWHM), indicated by the black arrows, remains constant within ±1 MHz. The PL brightness *Φ* increases threefold for 20 nm FNDs inside the cavity, while it decreases by about half for the 100 nm FNDs. In both cases, however, we observe spectrally narrower PL in the NV^−^ spectral region between 650 and 700 nm (20 nm FNDs) and 700 and 800 nm (100 nm FNDs) for FNDs inside the cavity (see Figure S9, Supporting Information). Spectral narrowing likely plays a dominant role in the enhancement of ODMR contrast observed in both samples' cavity regions and suggests that the NV coupling to the cavity can selectively enhance NV^−^ in our cavities.

Based on these measurements, we calculated the FND magnetic field sensitivity S using^[^ ^55^ ^]^

(4) $$S=\frac{4}{3\sqrt{3}}\frac{h}{g_{e}\mu_{B}}\frac{\Delta \nu }{C\sqrt{R}}$$

where *h* is Planck's constant, *g*_*e*_ ≈ 2.003 is the NV^−^ centers electronic g‐factor, *µ*_*B*_ is the Bohr magneton, *Δν* is the FWHM of the ODMR spectrum determined via a double Lorentzian fit, *C* is the ODMR contrast, and *R* the PL photon count rate. Figure 4d shows *S* for 20 and 100 nm FNDs inside and outside the cavity. It reveals a 4.8‐fold improvement in sensitivity for 20 nm FNDs and a 1.4‐fold improvement for 100 nm FNDs. The 4.8‐fold improvement for the 20 nm FNDs is driven by a combination of increase in ODMR contrast and PL brightness.

To investigate the trade‐off between cavity enhancement and sensor proximity to the sensing target, we estimated the magnetic field *B* at the location of the FND sensor as a function of its distance from a source (e.g., an electrical current in a microchip). Figure 4e shows $B\left(d\right)=\frac{S}{d/d_{0}}$, where *S* = 1 µT (the smallest magnetic field the cavity‐enhanced 20 nm FNDs can detect per $\sqrt{Hz}$) and *d* is the distance between sensor and sensing target. The orange trace represents *d*_0_ = *L*/2 + *T* = 104 nm, which is the distance between 20 nm FNDs and the sensing target in our devices. The markers indicate the smallest B‐field detectable per $\sqrt{Hz}$ for 20 nm FNDs inside and outside the cavity in our devices. For a hypothetical thicker cavity with *d*_0_ = 495 nm (blue trace in Figure 4e) and assuming the same 4.8‐fold enhancement observed for our devices, the cavity enhancement would not offer any benefits over the thinner no‐cavity devices investigated here. Hypothetical thicker cavities will need to create stronger enhancements and have lower optical losses to compensate for the larger separation between FND sensor and the sensing target.

While standoff significantly influences sensitivity, it has a relatively minor impact on spatial resolution for standoff distances of a few hundred nanometers. Considering the standoff distance between a potential sensing target and FNDs in our cavities (*T* ≈ 104 nm), we estimate a spatial resolution of approximately 156 nm (see Supporting Information for further details). This is well below the diffraction limit of our current optical setup (≈590 nm), so optical diffraction, rather than FND standoff, determines the practical resolution. For the envisaged application of magnetic imaging in microcircuits, typical surface‐accessible feature spacings are roughly two orders of magnitude larger than this estimated resolution. Therefore, the trade‐off between cavity thickness and cavity enhancement, rather than resolution, will guide the design of future thin‐film cavity devices using low‐loss cavity materials.

## 3 Conclusion

We have demonstrated cavity‐mediated emission enhancement in a suite of cavity devices containing fluorescent nanodiamonds and hexagonal boron nitride nanoparticles, underscoring the efficacy of Fabry–Pérot microcavities in improving the fluorescence properties of this class of quantum sensors. We find that the NV spectral emission peak position of FNDs in thin film cavities closely tracks the cavity resonance position and can be efficiently tuned between 600 and 730 nm via the polymer layer thickness. The spectral emission profile of FNDs inside the cavity becomes narrower compared to FNDs outside the cavity, allowing a selective enhancement of NV^−^ emission. The NV PL lifetime of FNDs inside cavity devices shortens by up to a factor of three and the observed total PL brightness remains largely unchanged, suggesting a Purcell enhancement‐induced increase in the NV radiative decay rate. We find a 4.8‐fold improvement of the ODMR magnetic field sensitivity of 20 nm FNDs inside thin film cavities due to an improved ODMR contrast.

The PL brightness of hBN particles is significantly enhanced in all cavity devices, but the hBN spectral emission properties are not efficiently modulated by the cavity resonances. At the same time, the observed PL decay rate increases by up to 13‐fold. This increase is significantly higher than the theoretical Purcell enhancement factor calculated for our devices, suggesting other enhancement mechanisms, such as plasmon‐related near‐field effects and electron transfer between hBN and the metal mirrors. We were unable to obtain ODMR spectra for hBN NPs within thin‐film cavities. This could be caused by the attenuation of the MW field by the cavity mirrors, which would further reduce the already low ODMR contrast of ∼1% commonly observed for the hBN material used here. Another explanation is that the PL lifetime of the hBN emitters inside the cavity observed here was too short for transitions to the metastable state to be efficient, resulting in no ODMR contrast. Overall, the observed enhancements in emission rates and ODMR signal contrast for FNDs suggest significant potential for thin‐film cavities in quantum sensing applications, enabling wafer‐scale fabrication of enhanced quantum sensors for optimized device integration.

## 4 Experimental Section

### Sample Preparation

FNDs were fabricated by irradiating HPHT nanodiamonds (120 nm, Nabond, China) with 2 MeV electrons to a fluence of 1 × 10^18^ electrons cm^−2^, then annealing them in argon at 900 °C for 2 h, and oxidizing the particle powder in air at 520 °C for 2.5 h. Nanoparticles of hBN (Graphene supermarket, 70 nm particle size) were used as‐received. Microcavity devices were fabricated by subsequent thin‐film deposition. First, 100 nm silver (Kurt J. Lesker PVD‐75 Proline with Electron Beam) was deposited by physical vapor deposition, with a 10 nm chromium adhesion layer. Then, quantum sensor‐doped polymer films were deposited by spin coating. PVP was used as the host polymer for FNDs and PMMA was used as the host polymer for hBN. Finally, a 25 nm silver mirror was deposited by physical vapor deposition to complete the cavity. For optical characterization, 20 × 20 mm^2^ polished silicon substrates were used. For quantum sensing characterization, 10 × 10 mm^2^ quartz substrates were used to minimize MW absorption by the substrate.

### Reflectance Microscopy

Normal‐angle reflectance measurements were acquired through a 20×, 0.45 NA optical air objective (Nikon, Japan) with light light irradiation from a xenon lamp (Nikon, Japan). Reflectance signal was recorded by a fiber‐coupled spectrometer (Ocean Optics, USA).

### Confocal Microscopy

Time‐resolved and steady‐state photoluminescence spectroscopy was performed using a custom‐built confocal microscope. In all measurements, 520 ± 10 nm ps‐pulsed laser (WhiteLase, Fianium, UK) excitation was delivered through a 100× objective (TU Plan Apo EPI, NA = 0.90, Nikon, Japan). Fluorescence was separated from the excitation path using a 532 nm dichroic mirror (Semrock, USA), followed by spectral filtering through a 540 nm long‐pass filter (Thorlabs, USA). Photoluminescence photons were split by a 75:25 multimode fiber beamsplitter: 25% of photons were detected with an avalanche photodiode (Excelitas, USA) and analyzed with a correlator card (Picoquant, Germany) for time‐resolved decay traces, and 75% of photons were detected with a spectrometer (Princeton Instruments, USA) for steady‐state spectra. Confocal photoluminescence maps were acquired with a piezoelectric scanning stage (Princeton Instruments, USA).

### Quantum Microscopy

The microscope utilized for ODMR cavity measurements is illustrated in Figure S6 (Supporting Information). Excitation is provided by a 532 nm continuous‐wave laser (Opus 2 W, Laser Quantum, Lastek, Australia), passed through a 400 mm convergent lens and a 538 nm dichroic mirror (Semrock, USA), and was focused on the back aperture of a 20×, NA = 0.45 objective (S PLAN Fluor, Nikon, Japan). This results in a ∼50 µm beam diameter at the sample with a central power density of ∼0.5 mW µm^−2^. Fluorescence was collected through the same objective and dichroic, collimated by a *f* = 300 mm convergent lens, and filtered sequentially with a 550 nm long‐pass and a 900 nm short‐pass filter (Thorlabs, USA) before detection on a CMOS camera (Zyla 5.5‐W USB3, Oxford Instruments, UK). A 670–770 nm LED, combined via a 50 mm lens, enabled bright‐field imaging for coarse focusing.

Microwave (MW) driving was provided by a signal generator (Synth NV pro, Windfreak Technologies, United States) gated with an IQ modulator (TRF37T05EVM, Texas Instruments, United States), amplified using a 50 W RF amplifier (HPA‐50W‐63+, Mini‐Circuits, United States), and syncronized with laser gating and camera acquisition using a pulse pattern generator (PulseBlasterESR‐PRO 500 MHz, SpinCore, United States). MWs were delivered either through a wire‐loop antenna positioned above Si‐based samples or via an exposed stripline waveguide PCB for samples fabricated on quartz substrates.

All quantum measurements represent an ensemble‐averaged signal from many particles within three 27 × 27 µm^2^ regions of interest.

### Statistical Analysis

Steady‐state and time‐resolved confocal PL spectroscopy were performed on 10 quantum sensor particles in each cavity and no‐cavity device (Figures 2 and 3). The average PL spectra shown in Figure 2a,d and Figures S2 and S4 (Supporting Information) were obtained by averaging all no‐cavity particles (*n* = 80 FNDs and *n* = 70 hBN NPs). Box‐and‐whisker plots in Figure 2b,e were constructed from the distributions of the 10 measured particles in each cavity and no‐cavity device. The horizontal line within each box denotes the mean, the box edges represent the upper and lower quartiles, and the whiskers indicate the minimum and maximum values of λ_*em*_; statistical outliers, defined as values exceeding 1.5 × the interquartile range, are shown as grey scatter points.

The shaded envelopes in Figure 3a,e represent the standard deviation of the decay traces across all 10 particles in each cavity and no‐cavity device. Error bars in Figure 3b,f correspond to the standard deviation of the average lifetime ratio for the 10 particles measured on the same device substrate. Similarly, error bars in Figure 3c,g indicate the standard deviation of the average brightness, while those in Figure 3d,h represent the standard deviation of the average Purcell factor for each cavity particle relative to the mean of the corresponding 10 no‐cavity particles on the same substrate. In Figure S7 (Supporting Information), error bars reflect the uncertainty associated with the double‐Lorentzian fit to the ODMR signal, and in Figure S10 (Supporting Information) they denote the standard deviation of the average brightness across 15 particles in each cavity and no‐cavity device.

All statistical analyses were completed using NumPy and/or SciPy libraries in Python, or IGOR Pro.

## Conflict of Interest

The authors declare no conflict of interest.

## Supporting information



Supporting Information

## Data Availability

The data that support the findings of this study are available from the corresponding author upon reasonable request.
