# Supplementary material for: A Scalable Method for Cavity‐Enhanced Solid‐State Quantum Sensors
Source: Adv Sci (Weinh). 2025 Dec 19;13(10):e17593. doi: 10.1002/advs.202517593 (PMC12915114; doi:10.1002/advs.202517593)
Supplement: Supplementary file 1 — Supporting Information [file ADVS-13-e17593-s001.pdf]

Supplementary Information for "A Scalable Method for Cavity–Enhanced Solid–State Quantum Sensors"

Daniel J. Tibben*,[*,†,‡] Roy Styles,[†,‡] David A. Broadway,[†] Jean-Philippe Tetienne,[†] Daniel E. Gómez*,[*,†] and Philipp Reineck*[*,†]

†*School of Science, RMIT University, Melbourne, Australia*

‡*These authors contributed equally to this work.*

E-mail: daniel.tibben@rmit.edu.au; daniel.gomez@rmit.edu.au; philipp.reineck@rmit.edu.au

Normal Angle Reflectance of FND and hBN Microcavity Devices

The normal angle reflectance spectra, displayed in Fig. S1, were used to determine the experimental resonance wavelength λ_{res} using Eq. S5 for the FND and hBN suites of devices.

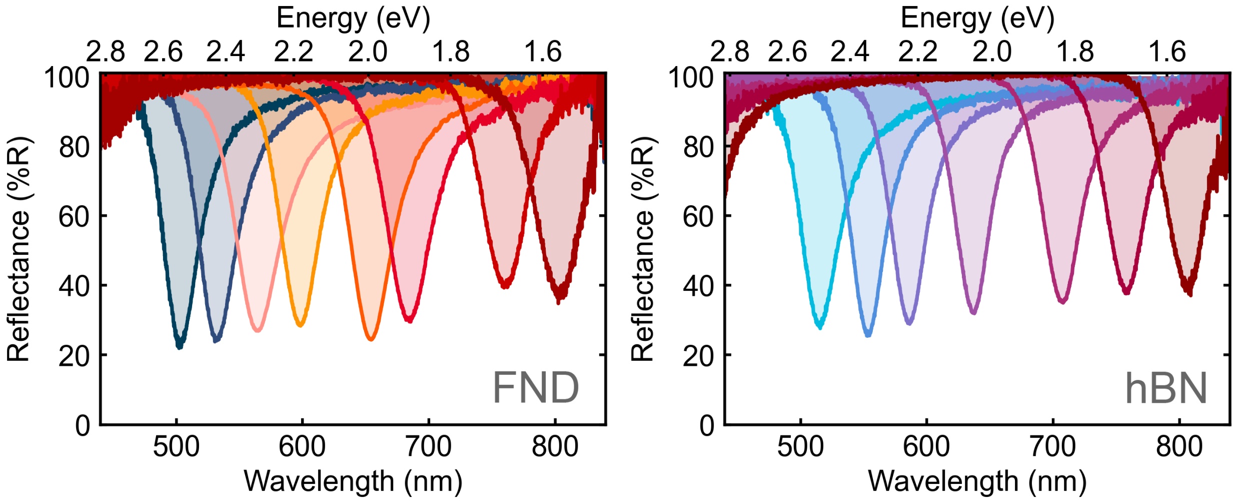

Figure S1: Normal angle reflectance of the cavity devices.

Transfer Matrix Model for Fabry–Pérot Microcavities

The resonance energies of the Fabry–Pérot microcavity were calculated using a transfer matrix model. In this approach, each optical layer of thickness d_i and refractive index $n_i(\omega)$ is represented by a propagation matrix, while interfaces between layers are described by Fresnel reflection and transmission coefficients. Multiplying these matrices yields the overall transfer matrix of the cavity. The reflectance spectrum is then obtained from the reflection amplitude $r(\omega)$, with $R(\omega) = |r(\omega)|^2$.

The transfer matrix for a single layer of refractive index n_i, thickness d_i, and wavevector component

$$k_i = \frac{2\pi}{\lambda} n_i \cos\theta_i \tag{S1}$$

(at internal angle θ_i) can be written as

$$M_i = \begin{bmatrix} \cos(k_i d_i) & \frac{i}{q_i}\sin(k_i d_i) \\ i q_i \sin(k_i d_i) & \cos(k_i d_i) \end{bmatrix}, \tag{S2}$$

where $q_i = n_i \cos\theta_i$ for transverse electric (TE) polarization or $q_i = \frac{\cos\theta_i}{n_i}$ for transverse magnetic (TM) polarisation. The total transfer matrix for the full cavity is then

$$M = \prod_i M_i, \tag{S3}$$

with boundary conditions applied at the air/cavity and cavity/substrate interfaces.

The Fabry–Pérot resonance condition corresponds to the round-trip phase in the cavity spacer, including mirror phase contributions, which can be simply modeled using

$$2n_{\text{eff}}L\frac{2\pi}{\lambda} + \phi_r = 2m\pi, \tag{S4}$$

where L is the polymer thickness, n_{eff} is its effective refractive index, ϕ_r is the cumulative phase shift upon reflection from the silver mirrors, and m is an integer mode index. Resonance energies $E = hc/\lambda$ therefore appear as minima in the reflectance spectrum, which can be directly compared with experimental reflectance dips. In unpolarized experiments, such as that in Fig. S1, the resonance wavelength λ_{res} is simply determined by

$$\lambda_{res} = 2n_{\text{eff}}L/m. \tag{S5}$$

For FND-doped films, polyvinyl pyrrolidone (PVP) was used, and for hBN-doped films, polymethyl methacrylate (PMMA) was used. Including the contributions from the top and bottom silver mirrors (and any contributions from the quantum sensor particles) the n_{eff} was determined to be 2.04 for PVP cavities and 1.98 for PMMA cavities.

Table S1: Modeled resonance wavelength of cavity devices fabricated on Si.

Device Type	Predicted Resonance λ_0 (nm)
PVP (FND-doped)	500
PVP (FND-doped)	532
PVP (FND-doped)	570
PVP (FND-doped)	600
PVP (FND-doped)	650
PVP (FND-doped)	700
PVP (FND-doped)	750
PVP (FND-doped)	800
PMMA (hBN-doped)	515
PMMA (hBN-doped)	560
PMMA (hBN-doped)	590
PMMA (hBN-doped)	640
PMMA (hBN-doped)	700
PMMA (hBN-doped)	750
PMMA (hBN-doped)	800

Purcell Enhancement in Fabry-Pérot Micocavities

To characterise the emission enhancement by the resonant conditions in the cavity, we describe our results in terms of the Purcell enhancement of both emission rates and brightness between the 'cavity' and 'no-cavity' devices.

The brightness Φ of our devices is defined as the number of photons emitted from the device across the collected spectral region. This is calculated by taking the integral of the spectral profile using the trapezoidal method:

$$\Phi = \int_{x_a}^{x_b} f(x)dx \approx \frac{x_b - x_a}{2N} \sum_{n=1}^{N} (f(x) + f(x_{n+1})) \tag{S6}$$

where x_a and x_b are the start and end wavelength of the spectral region.

The Purcell model we use to characterize the enhancement in our devices was developed by Englund et al. [1], and is described in Eq. 2.

The theoretical Purcell factor can be calculated for these devices by

$$F_P^{th.} = \frac{3\lambda_{res}^3 Q}{4\pi^2 n^3 V_{eff}} \tag{S7}$$

where λ_{res} is the cavity resonance wavelength, n is the refractive index of the cavity medium and V_{eff} is the effective mode volume in the cavity. In this case, we use an idealized approach and assume perfect longitudinal confinement in the planar cavity, with no lateral confinement, where the assumption $V_{eff} \simeq 0.125(\frac{\lambda}{n})^3$ is appropriate [2].

This reduces the theoretical Purcell factor calculation to

$$F_P^0 \simeq \frac{6Q}{\pi^2} \tag{S8}$$

which is a valid assumption for this class of Fabry-Pérot microcavity device, where the lateral physical dimension exceeds the longitudinal physical dimension by at least 5 orders of magnitude, resulting in dominated confinement in the longitudinal direction and negligible confinement in the lateral direction.

Cavity Q-factor Determination

The quality factor of the cavity devices was determined experimentally from the inverse reflectance spectra.

The cavity quality factor, Q, is defined as

$$Q = \frac{\lambda_0}{\Delta\lambda} \tag{S9}$$

where λ_0 is the resonance wavelength and $\Delta\lambda$ is the full width at half maximum (FWHM) of the inverse reflectance peak. In the experimental reflectance spectra, λ_0 was identified as the wavelength at maximum inverse reflectance. The FWHM, $\Delta\lambda$, was determined from the wavelengths at which the inverse reflectance crossed half of the peak amplitude relative to a

baseline. The baseline was calculated from the edges of the spectrum, and cubic interpolation was applied to the inverse reflectance data to determine the half-maximum crossing points accurately.

Table S2: List of theoretical maximum Purcell factors for fluorescent nanodiamond and hBN cavity devices based on cavity Q-factor .

**FND**			**hBN**		
Resonance (nm)	Q-factor	Purcell factor $F_P^{th.}$	Resonance (nm)	Q-factor	Purcell factor $F_P^{th.}$
500	13.02	7.92	515	11.76	7.15
532	11.57	7.03	560	13.48	8.19
570	12.62	7.67	590	14.06	8.55
600	12.85	7.81	640	15.72	9.56
650	15.95	9.70	700	20.66	12.56
700	12.72	7.73	750	23.82	14.48
750	15.39	9.36	800	19.21	11.68
800	21.34	12.97			
Median Value:	12.94	5.16	Median Value:	15.72	6.27

Cavity Enhancement Within a Gaussian Projection of the Cavity Mode

To extract a projection of the cavity mode on emission from the cavity, cavity emission spectra were analyzed by fitting a sum of two Gaussian functions,

$$I(\lambda) = A_1 \exp\left[-\frac{(\lambda - \mu_1)^2}{2\sigma_1^2}\right] + A_2 \exp\left[-\frac{(\lambda - \mu_2)^2}{2\sigma_2^2}\right], \tag{S10}$$

using nonlinear least–squares optimization. The cavity peak maximum was identified from the fit, and an integration range of ±15 nm was applied around this resonance.

The integrated cavity emission in this range was compared with the corresponding integrated intensity from reference (no cavity) spectra. No cavity reference areas were averaged across multiple particles for fair comparison. The cavity enhancement ratio η was defined as

$$\eta = \frac{\int_{\lambda_{\max}-15}^{\lambda_{\max}+15} I_{\text{cav}}(\lambda)\, d\lambda}{\left\langle \int_{\lambda_{\max}-15}^{\lambda_{\max}+15} I_{\text{no-cav}}(\lambda)\, d\lambda \right\rangle}. \tag{S11}$$

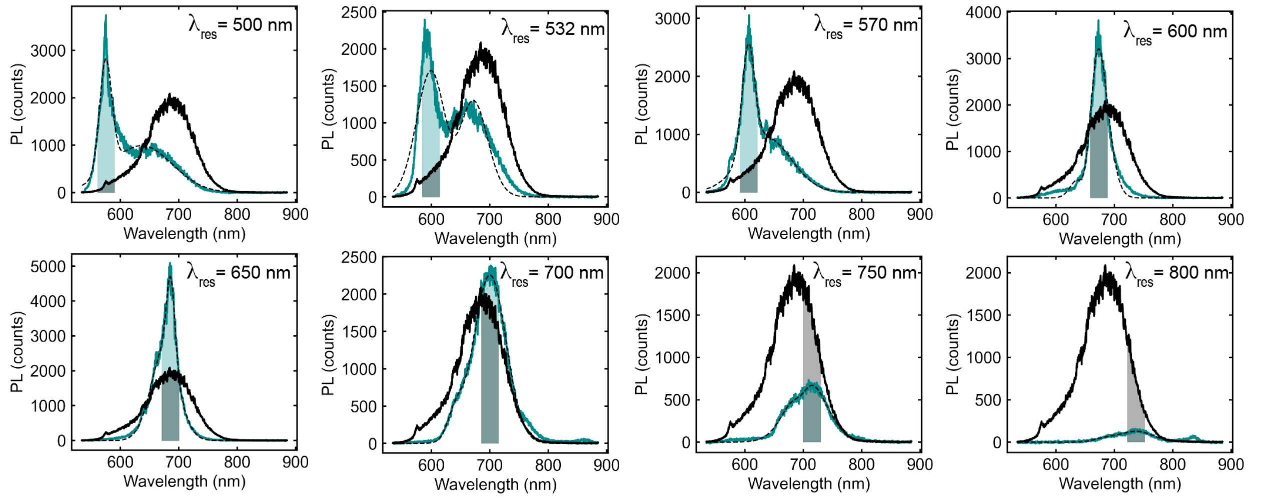

Figure S2: Fitting a double Gaussian projection (black dashed lines) of the cavity mode on example FND cavity emission spectra (teal line) and the average FND no cavity control emission spectrum (black line), as defined in Eq. S11.

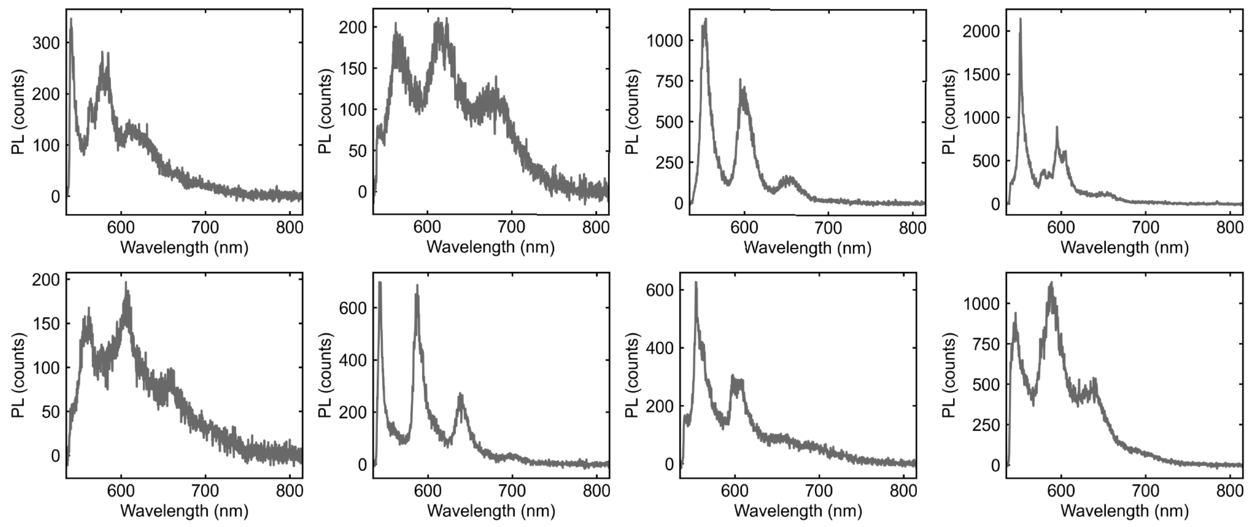

Figure S3: Photoluminescence variability in hBN particles outside the cavity.

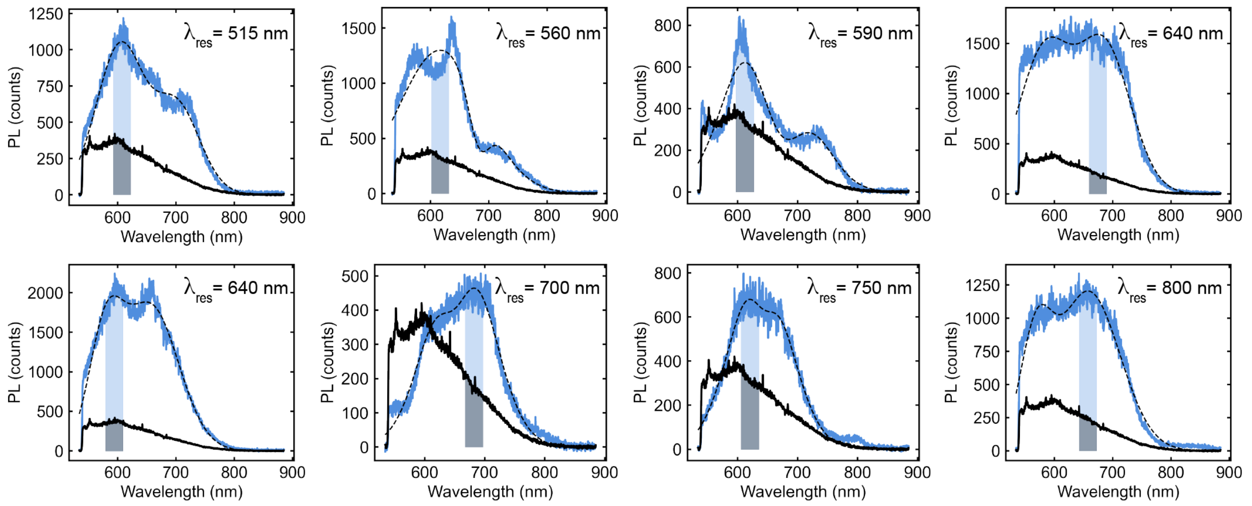

Figure S4: Fitting a double Gaussian projection (black dashed lines) of the cavity mode on example hBN cavity emission spectra (blue line) and the average hBN no cavity control emission spectrum (black line), as defined in Eq. S11. Emission maxima and lineshape of hBN cavity devices do not correlate well with the resonance energies determined from measured reflectance in Fig. S1.

hBN Emission Characteristics

The PL spectra of hBN nanoparticles are usually non-uniform due to the heterogeneous distribution of luminescent centers, and their surrounding environments. Emission typically originates from intrinsic point defects such as vacancies, donor–acceptor pairs, and impurity complexes, each associated with distinct ZPLs and phonon sidebands. Theoretical predictions [3, 4] and large-scale statistical studies [5] have shown that hBN emitters can be grouped into several discrete spectral families within the $\sim 1.6 - 2.2$ eV range, indicating the coexistence of multiple defect types even within nominally identical samples. Additionally, local variations in dielectric screening, strain, and surface termination can lead to shifts in emission energies and differences in spectral line shapes. These effects are further influenced by particle size, morphology, and interactions with substrates or the surrounding environment.

Non-uniformity is also affected by extrinsic contributions from organic residues, including aromatic polycyclic hydrocarbons introduced during processing and have been reported to act as additional luminescent centers in some hBN samples [6]. These emitters contribute their own PL features and complicate the spectral response of the nanoparticles. Temporal

instabilities such as blinking and bleaching, which are common in nanoscale fluorophores due to charge trapping or non-radiative recombination, further add to spectral variability. These intrinsic and extrinsic effects account for the observed unpredictability in the PL spectra of hBN nanoparticles and highlight the challenges of achieving reproducible optical performance.

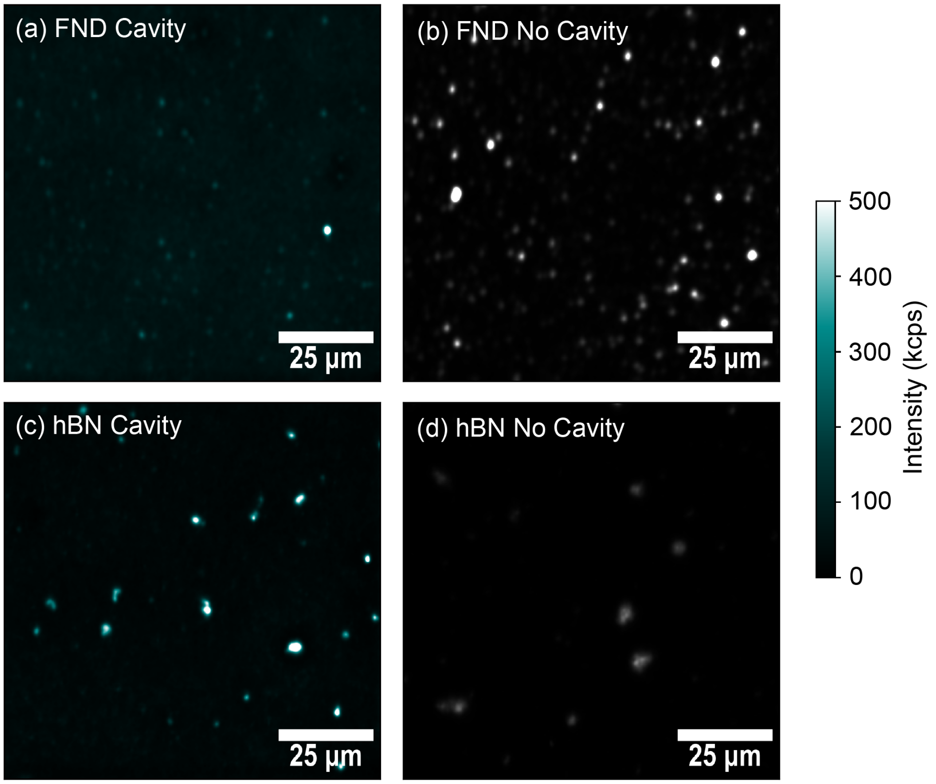

Figure S5: Confocal photoluminescence maps of quantum sensors in cavity and no cavity devices. (a) FNDs in the $\lambda_{res} = 650$ nm cavity device, (b) FNDs in no cavity device on the same substrate, (c) hBN NPs in the $\lambda_{res} = 640$ nm cavity device, and (d) hBN NPs in no cavity device on the same substrate.

Exponential Decay Model

To characterize the temporal decay dynamics of the emission, the time-resolved photoluminescence data were fitted using a bi-exponential model of the form

$$I(t) = A_1 \exp\left(-\frac{t}{\tau_1}\right) + A_2 \exp\left(-\frac{t}{\tau_2}\right), \tag{S12}$$

where A_1 and A_2 are the relative amplitudes, and τ_1 and τ_2 are the characteristic lifetimes of the fast and slow decay channels, respectively. This second-order exponential fit accounts for the presence of multiple recombination pathways or emissive states within the system, which cannot be adequately described by a single exponential. From the fitted parameters, an amplitude weighted average lifetime was calculated according to

$$\langle \tau \rangle = \frac{A_1 \tau_1 + A_2 \tau_2}{A_1 + A_2}, \tag{S13}$$

which provides a representative metric for comparison across different spectra. This average lifetime captures the effective emission dynamics of the ensemble, while preserving sensitivity to both short- and long-lived components of the decay process.

Quantum Microscope

The microscope utilized for quantum measurements is described in the methods section of the main text and illustrated below in Figure S6.

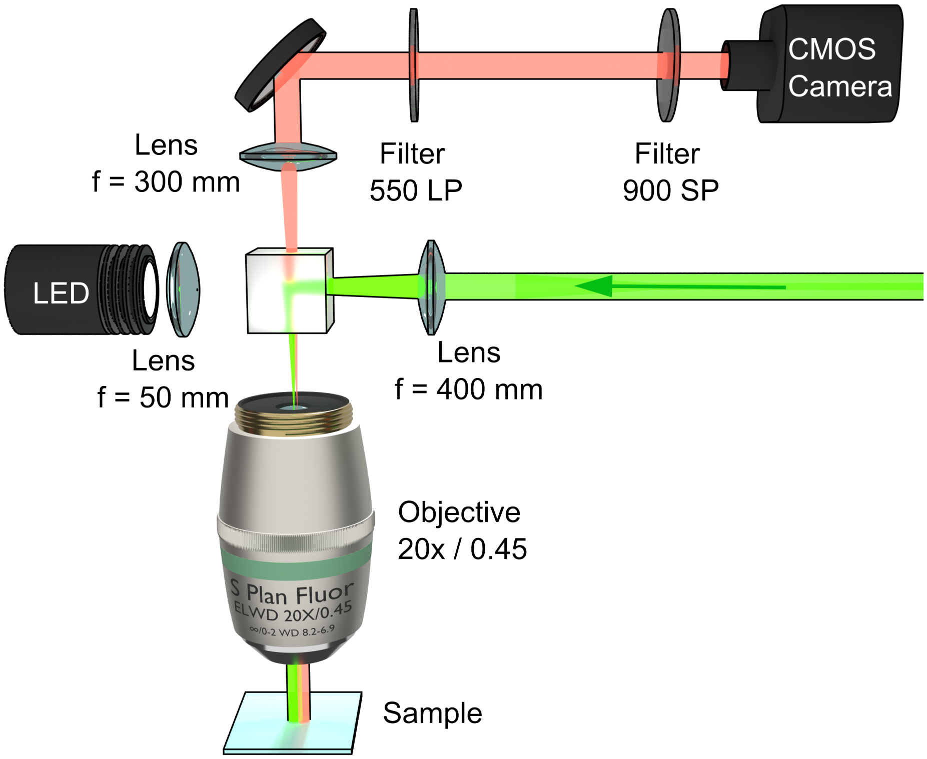

Figure S6: Schematic of the wide-field microscope used for fluorescence and bright-field imaging. A 532 nm excitation laser is focused into the back aperture of a 20×/0.45 NA objective. Emission is collected through the same objective, collimated with a 300 mm lens, spectrally filtered (500 nm LP, 600 nm LP, 850 nm SP), and imaged onto a CMOS camera. An LED coupled via a 50 mm lens and beamsplitter provides bright-field illumination for coarse alignment, while microwaves are delivered either via a wire loop or a PCB stripline depending on the substrate.

ODMR Contrast of Cavities Fabricated on Silicon

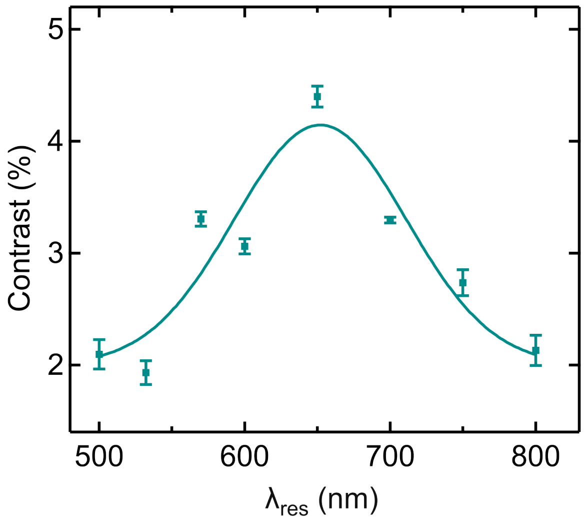

Figure S7: ODMR contrast of 120 nm FNDs in Si-substrate-cavities as a function of cavity resonance wavelength. Markers represent the ODMR contrast determined from averaged spectra across three regions of interest (125 × 125 μm) via a double Lorentzian fit. Error bars denote the fit uncertainty. The solid trace is a Gaussian fit to the data and a guide to the eye only.

ODMR spectra for 120 nm FNDs in a cavities fabricated on Si substrates were acquired using the quantum microscope described in the preceding section. MW driving was supplied via a wire loop antenna to minimize absorption losses in the Si substrates. For each cavity, ODMR spectra were acquired for three regions of interest (125 x 125 µm) and averaged. Figure S7 plots the extracted ODMR contrast (markers) as a function of resonance wavelength. Each data point was obtained by fitting the ODMR spectrum to a Lorentzian function, with error bars representing the uncertainty in the fit.

Normal Angle Reflectance Spectra of Cavity Devices on Quartz Substrates for Quantum Sensing

Normal angle reflectance spectra, used to determine the experimental λ_{res} values for quantum sensing devices fabricated on quartz substrates, are displayed in Fig. S8. Measured vs. predicted peak positions are displayed in Table S3.

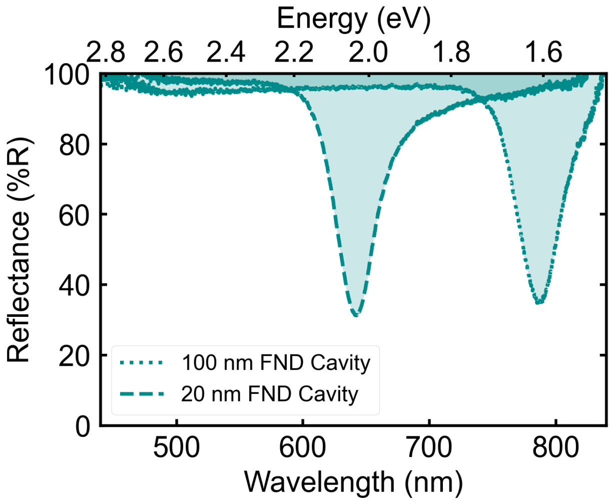

Figure S8: Normal angle reflectance spectra of the quantum sensing cavity devices.

Table S3: Cavity device or quartz parameters for quantum sensing. Predicted resonance is based on the transfer matrix model in Eq. S3, and actual resonance is determined according to the approximation in Eq. S5.

Device Type	Predicted Resonance λ_0 (nm)	Actual Resonance λ_{res} (nm)
PVP (100 nm FND-doped)	650	788
PVP (20 nm FND-doped)	650	642

FND Spectra from Cavities Fabricated on Quartz

Average PL spectra from FNDs inside and outside the cavity regions on quartz substrates are shown in Figure S9. The 20 nm FNDs (Fig. S9a) exhibited a pronounced enhancement in PL brightness within the cavity regions, particularly in the NV^- phonon sideband region. To obtain these spectra, PL spectra were acquired continuously during the acquisition of a 20 x 20 µm confocal scan and averaged. This method was utilized because the particle density of 20 nm FNDs was high enough that averaging over the scanned region yielded a representative spectrum without the need to isolate individual particles. In contrast, the 100 nm FNDs (Fig. S9b) exhibited a narrowing of the emission spectra with an emission peak in the 700-800 nm region corresponding to the measured cavity resonance wavelength, as detailed in Table S3, accompanied by a suppression of the PL at shorter wavelengths.

Due to the lower density of 100 nm FNDs on the substrate, spectra were acquired by scanning a 20 x 20 µm region and selecting 15 individual particles at random. These individual spectra were then averaged.

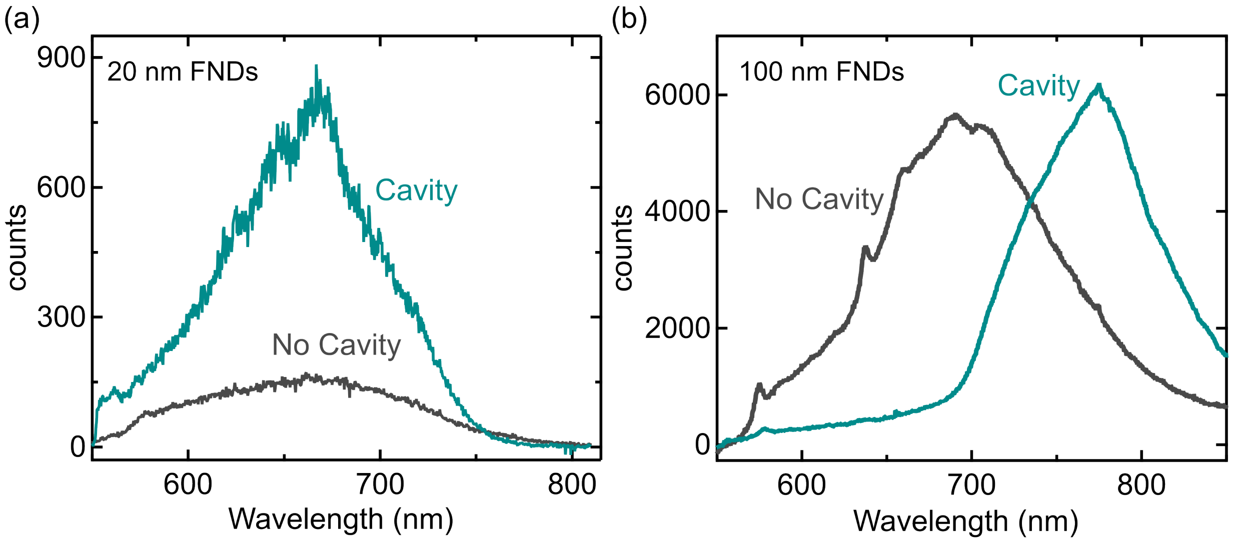

Figure S9: Spectra from particles inside (teal) and outside (grey) cavity regions for the 20 nm particles and 100 nm particles on quartz substrates. Both data sets were acquired using a confocal scanning microscope and scanning a 20 x 20 µm randomly selected region. (a) Spectra from the 20 nm particles were acquired by conducting a long exposure of the spectrometer for the entire duration of the 20 x 20 µm scans. (b) Spectra for the 100 nm particles were acquired by scanning a 20 x 20 µm region and acquiring the spectra of 15 particles at random and averaging them together.

FND Brightness from Cavities Fabricated on Quartz

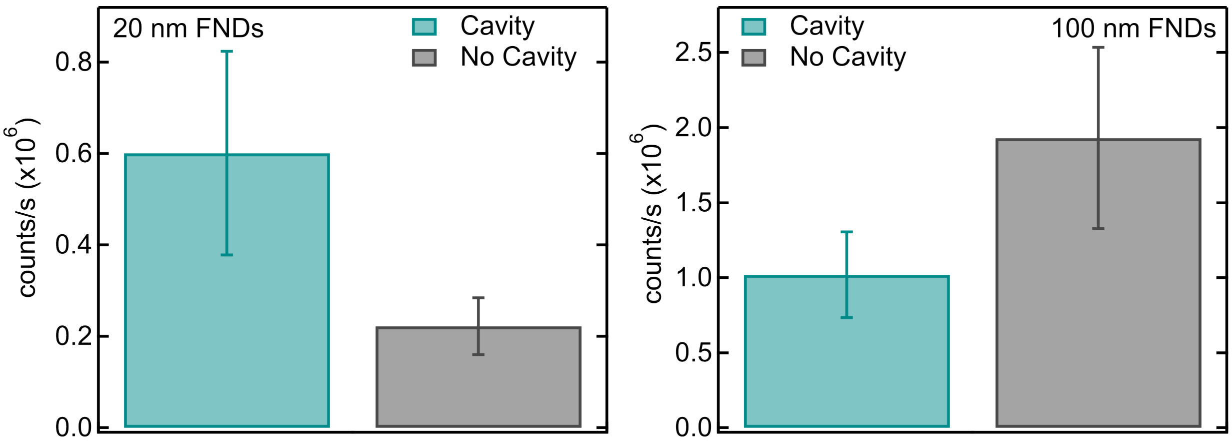

Figure S10: Brightness of particles inside (teal) and outside (grey) cavity regions for the 20 nm particles and 100 nm particles on quartz substrates. Data was acquired with a confocal scanning microscope by scanning a randomly selected region and observing the brightness of 15 particles each. The brightness of FND's were averaged together. The error bars show the standard deviation in the averaged FND brightnesses.

The brightness of particles inside and outside of cavities was measured using a scanning confocal microscope with a 532 nm excitation laser at 300 µW. On both samples a random region (20 x 20 µm) was scanned on the cavity side and on the no cavity side, resulting in four scanned regions. From each scanned image, the brightness of 15 particles was measured and compared. On average 20 nm FNDs in the cavity were 2.7x brighter at 6×10^5 counts/s compared to those outside of the cavity. For the 100 nm FNDs the opposite trend was observed, being 0.4x dimmer inside the cavity than those outside. This result coincides with the observed spectral data in Fig. S9, which shows that while the 20 nm FNDs had a large enhancement in brightness across the NV phonon sideband (PSB), the 100 nm enhancement resulted in a narrowing of the emission spectra in the PSB accompanied by a quenching of the PL at lower wavelengths. As a result, dimming from absorption and reflectance at the top cavity mirror may have a more significant effect in the 100 nm samples, where no clear broadband increase in PL intensity was observed. Thus, the apparent reduction in brightness of the 100 nm FNDs can be attributed to a combination of spectral redistribution

and cavity-induced optical losses.

Spatial Resolution of FNDs in Quartz Cavities

The spatial magnetic resolution can be estimated using the point-spread function (PSF) for in-plane magnetic field components [7]:

$$a_{x,y} = \frac{d}{2\pi(\rho^2 + d^2)^{\frac{2}{3}}} \tag{S14}$$

Here, $a_{x,y}$ describes the lateral blurring (as a PSF) of a magnetic feature (at point $\rho = 0$) as its field propagates to an NV centre located at a height d. Solving for the FWHM of the PSF gives a spatial resolution of $\Delta x \approx 1.5d$. In the quartz cavities utilised in Fig. 4, assume an NV in the centre of a 20 nm FND. We can define $d = T_{Ag} + 0.5T_{PVP} = 104$ nm, therefore $\Delta x \approx 156$ nm. While features down to 156 nm of separation should be magnetically resolvable by individual particles, the current diffraction limit of ≈ 590 nm (NA 0.45, $\lambda = 532$ nm) prevents individual readout of particles that are closer together.

Background Polymer Fluorescence in Polyvinyl Pyrrolidone

PVP becomes slightly fluorescent in the visible after exposure to heat in the electron beam physical vapor deposition process. This emission readily photobleaches and is much dimmer than the emission from FNDs. PMMA shows no such fluorescence in the visible.

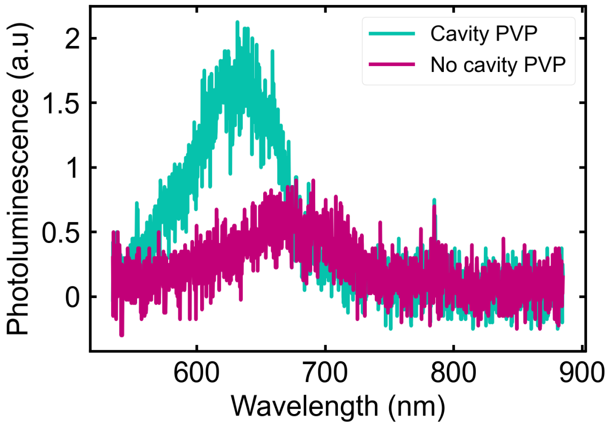

Figure S11: PVP polymer emission from the bare film inside and outside the cavity for a 650 nm resonant cavity.

References

(1) Englund, D.; Shields, B.; Rivoire, K.; Hatami, F.; Vučković, J.; Park, H.; Lukin, M. D. Deterministic Coupling of a Single Nitrogen Vacancy Center to a Photonic Crystal Cavity. *Nano Letters* **2010**, *10*, 3922–3926.

(2) Gérard, J.-M. *Single Quantum Dots: Fundamentals, Applications, and New Concepts*; Springer: Berlin, Heidelberg, 2003; pp 269–314.

(3) Sajid, A.; Reimers, J. R.; Ford, M. J. Defect states in hexagonal boron nitride: Assignments of observed properties and prediction of properties relevant to quantum computation. *Physical Review B* **2018**, *97*, 064101, Publisher: American Physical Society.

(4) Auburger, P.; Gali, A. Towards ab initio identification of paramagnetic substitutional carbon defects in hexagonal boron nitride acting as quantum bits. *Physical Review B* **2021**, *104*, 075410, Publisher: American Physical Society.

(5) Islam, M. S.; Chowdhury, R. K.; Barthelemy, M.; Moczko, L.; Hebraud, P.; Berciaud, S.; Barsella, A.; Fras, F. Large-Scale Statistical Analysis of Defect Emission in

hBN: Revealing Spectral Families and Influence of Flake Morphology. *ACS Nano* **2024**, *18*, 20980–20989, Publisher: American Chemical Society.

(6) Neumann, M.; Wei, X.; Morales-Inostroza, L.; Song, S.; Lee, S.-G.; Watanabe, K.; Taniguchi, T.; Götzinger, S.; Lee, Y. H. Organic Molecules as Origin of Visible-Range Single Photon Emission from Hexagonal Boron Nitride and Mica. *ACS Nano* **2023**, *17*, 11679–11691, Publisher: American Chemical Society.

(7) Casola, F.; van der Sar, T.; Yacoby, A. Probing Condensed Matter Physics with Magnetometry Based on Nitrogen-Vacancy Centres in Diamond. *Nature Reviews Materials* **2018**, *3*, 17088.
